# Geostatistical Model-Based Estimates of Schistosomiasis Prevalence among Individuals Aged ≤20 Years in West Africa

**DOI:** 10.1371/journal.pntd.0001194

**Published:** 2011-06-14

**Authors:** Nadine Schur, Eveline Hürlimann, Amadou Garba, Mamadou S. Traoré, Omar Ndir, Raoult C. Ratard, Louis-Albert Tchuem Tchuenté, Thomas K. Kristensen, Jürg Utzinger, Penelope Vounatsou

**Affiliations:** 1 Department of Epidemiology and Public Health, Swiss Tropical and Public Health Institute, Basel, Switzerland; 2 University of Basel, Basel, Switzerland; 3 Programme National de Lutte Contre la Bilharziose et les Géohelminthes, Ministère de la Santé Publique et de la Lutte Contre les Endémies, Niamey, Niger; 4 Institut National de Recherche en Santé Publique, Ministère de la Santé, Bamako, Mali; 5 Service de Parasitologie, Faculté de Médecine, Pharmacie et Odontologie, Université Cheikh Anta Diop, Dakar, Sénégal; 6 Office of Public Health, Louisiana Department of Health and Hospitals, New Orleans, Louisiana, United States of America; 7 National Programme for the Control of Schistosomiasis and Intestinal Helminthiasis, Ministry of Public Health, Yaoundé, Cameroon; 8 Laboratoire de Biologie Générale, Université de Yaoundé I, Yaoundé, Cameroon; 9 Centre for Schistosomiasis and Parasitology, Yaoundé, Cameroon; 10 DBL, Department of Veterinary Disease Biology, University of Copenhagen, Frederiksberg, Denmark; London School of Hygiene & Tropical Medicine, United Kingdom

## Abstract

**Background:**

Schistosomiasis is a water-based disease that is believed to affect over 200 million people with an estimated 97% of the infections concentrated in Africa. However, these statistics are largely based on population re-adjusted data originally published by Utroska and colleagues more than 20 years ago. Hence, these estimates are outdated due to large-scale preventive chemotherapy programs, improved sanitation, water resources development and management, among other reasons. For planning, coordination, and evaluation of control activities, it is essential to possess reliable schistosomiasis prevalence maps.

**Methodology:**

We analyzed survey data compiled on a newly established open-access global neglected tropical diseases database (i) to create smooth empirical prevalence maps for *Schistosoma mansoni* and *S. haematobium* for individuals aged ≤20 years in West Africa, including Cameroon, and (ii) to derive country-specific prevalence estimates. We used Bayesian geostatistical models based on environmental predictors to take into account potential clustering due to common spatially structured exposures. Prediction at unobserved locations was facilitated by joint kriging.

**Principal Findings:**

Our models revealed that 50.8 million individuals aged ≤20 years in West Africa are infected with either *S. mansoni*, or *S. haematobium*, or both species concurrently. The country prevalence estimates ranged between 0.5% (The Gambia) and 37.1% (Liberia) for *S. mansoni*, and between 17.6% (The Gambia) and 51.6% (Sierra Leone) for *S. haematobium*. We observed that the combined prevalence for both schistosome species is two-fold lower in Gambia than previously reported, while we found an almost two-fold higher estimate for Liberia (58.3%) than reported before (30.0%). Our predictions are likely to overestimate overall country prevalence, since modeling was based on children and adolescents up to the age of 20 years who are at highest risk of infection.

**Conclusion/Significance:**

We present the first empirical estimates for *S. mansoni* and *S. haematobium* prevalence at high spatial resolution throughout West Africa. Our prediction maps allow prioritizing of interventions in a spatially explicit manner, and will be useful for monitoring and evaluation of schistosomiasis control programs.

## Author Summary

Schistosomiasis is a parasitic disease caused by a blood fluke that mainly occurs in Africa. Current prevalence estimates of schistosomiasis are based on historical data, and hence might be outdated due to control programs, improved sanitation, and water resources development and management (e.g., construction of large dams and irrigation systems). To help planning, coordination, and evaluation of control activities, reliable schistosomiasis prevalence estimates are needed. We analyzed compiled survey data from 1980 onwards for West Africa, including Cameroon, focusing on individuals aged ≤20 years. Bayesian geostatistical models were implemented based on environmental and climatic predictors to take into account potential spatial clustering within the data. We created the first smooth data-driven prevalence maps for *Schistosoma mansoni* and *S. haematobium* at high spatial resolution throughout West Africa. We found that an estimated 50.8 million West Africans aged ≤20 years are infected with schistosome blood flukes. Country prevalence estimates ranged between 0.5% (in The Gambia) and 37.1% (in Liberia) for *S. mansoni* and between 17.6% (in The Gambia) and 51.6% (in Sierra Leone) for *S. haematobium*. Our results allow prioritization of areas where interventions are needed, and to monitor and evaluate the impact of control activities.

## Introduction

Schistosomiasis is a water-based disease caused by trematodes of the genus *Schistosoma*. The five schistosome species that are known to infect humans are *Schistosoma mansoni*, *S. haematobium*, *S. intercalatum*, *S. mekongi*, and *S. japonicum*. School-aged children are at highest risk of infection and are the main target group for interventions [1].

Despite successful efforts to control schistosomiasis in different parts of the world, more than 200 million individuals are still estimated to be infected and the annual global burden due to schistosomiasis might exceed 4.5 million disability-adjusted life years (DALYs) lost [1]–[3]. A substantial amount of this burden is concentrated in West Africa, including Cameroon. Indeed, 72 million infections are thought to occur in this part of the world [4]. However, the current statistics, as presented by Chitsulo *et al.* (2000) [4], Steinmann *et al.* (2006) [2], and Utzinger *et al.* (2009) [5], are largely based on population re-adjusted data originally published by Utroska and colleagues in the late 1980s [6]. Hence, the estimates are likely to be outdated due to, among other reasons, large-scale preventive chemotherapy campaigns, improved sanitation, water resources development and management, and socio-economic development.

Recently, donors have provided new funds to control the so-called neglected tropical diseases (NTDs), including schistosomiasis. For cost-effective planning and evaluation of control activities, it is essential to have reliable baseline maps of the geographical distribution of at-risk population and disease burden. Early schistosomiasis mapping efforts have been based on climatic suitability thresholds [7], [8]. These maps are not reliable because they are not based on disease data. Apart from a few studies [9]–[12], empirical maps of disease distribution over large areas are not available since there is a paucity of contemporary large-scale survey data.

The first comprehensive compilation of historical schistosomiasis prevalence surveys at a global scale was carried out by Doumenge *et al.* in the mid-1980s [13]. More recent collections are available by Brooker *et al.* (2010) [14] for soil-transmitted helminthiasis and schistosomiasis, but data access is limited. The European Union (EU)-funded CONTRAST project initiated the development of an open-access global NTD database, which is updated in real time (GNTD database; http://www.gntd.org) [15]. A key objective of CONTRAST is to employ this database for large-scale schistosomiasis prevalence mapping and prediction in sub-Saharan Africa for the spatial refinement of control interventions and the cost-effective allocation of resources.

Geographical locations in close proximity share common exposures which influence the disease outcome similarly. The geographical information of the survey locations in the GNTD database allows taking into account the potential spatial correlation and therefore creation of more realistic models. Standard statistical modeling approaches assume independence between locations [16]. Ignoring potential spatial correlation in neighboring areas due to common exposures could result in incorrect model estimates [17]. Geostatistical models take into account spatial clustering by introducing location-specific random effect parameters in the covariance matrix by a function of distance between locations [16]. Such models typically contain large numbers of parameters and cannot be estimated by the commonly used maximum likelihood approaches [18]. Bayesian model formulations enable model fit via Markov chain Monte Carlo (MCMC) simulations [16].

Bayesian geostatistical models have been applied in mapping schistosomiasis at different spatial scales, for example by Raso *et al.* (2005) [19] in the region of Man, western Côte d'Ivoire, and Clements *et al.* (2008) [9] in Mali, Niger, and Burkina Faso. Brooker *et al.* (2010) [14] developed a global predictive map highlighting those areas where preventive chemotherapy against schistosomiasis and soil-transmitted helminthiasis are warrant. However, to our knowledge, there is neither a model-based *S. haematobium* nor a *S. mansoni* large-scale prevalence map and spatially explicit burden estimates for the whole West African region.

In this paper, we developed Bayesian geostatistical models based on environmental and climatic risk factors to obtain reliable empirical schistosomiasis prevalence maps for individuals aged ≤20 years by analyzing the GNTD data for West Africa, including Cameroon. Prediction was based on joint kriging in order to summarize the results as population-adjusted country prevalence estimates. Emphasis was placed on the distribution of *S. haematobium* and *S. mansoni*. We neglected *S. intercalatum* due to low infection risks, especially outside Cameroon.

## Methods

### Disease data

The GNTD database was used to obtain prevalence data on schistosomiasis. This database assembles general information about the type of publication, authors, and publication year, as well as study-specific information about survey population, survey period, *Schistosoma* species, diagnostic test employed, and the number of infected individuals among those examined, stratified by age and sex (if available). Hospital studies, data on specific susceptible groups (such as HIV positives), and post-intervention studies were not included in the database [15]. For this study, we analyzed all point-level data on settled populations in West Africa on either *S. haematobium* or *S. mansoni*: 4550 and 2611 survey locations, respectively. We excluded (i) surveys with missing geographical coordinates; (ii) missing numbers of individuals screened; (iii) surveys carried out before 1980; (iv) individuals aged >20 years; and (v) entries based on certain diagnostic techniques. With regard to the latter exclusion criteria, we rejected all non-direct diagnostic examination techniques, such as immunofluorescence tests, antigen detections or questionnaire data, and direct fecal smears that have very low diagnostic sensitivities (overall, 4% of the data for *S. mansoni* and 0.1% for *S. haematobium* were excluded). Hence, the surveys included were mainly based on the Kato-Katz thick smear method (*S. mansoni*) and urine filtration or sedimentation (*S. haematobium*). Sensitivity and specificity of the diagnostic techniques were not incorporated in the model due to usually unknown sampling effort (e.g., number of stool samples, number of slides examined under a microscope, etc.), which affect diagnostic accuracy.

We assumed that the proportion of rejected diagnostic techniques among the data with missing information on the technique (*S. mansoni*: 33.5% missing, *S. haematobium*: 20.6% missing) is similar. Therefore, we considered the bias that would arise from ignoring the missing data as larger than the bias from potentially rejected diagnostic techniques among the missing data. A separate model validation on the reduced datasets confirmed that by including data with incomplete records the predictive ability increased compared to the model excluding this information (results not presented).

### Climatic, environmental, and population data

Climatic, environmental, and population data were obtained from different freely accessible remote sensing data sources, as summarized in Table 1. Data on day and night temperature were extracted from land surface temperature (LST) data. The normalized difference vegetation index (NDVI) was used as a proxy for vegetation. Digitized maps on freshwater body sources (e.g., rivers, lakes, and wetlands) in West Africa were acquired with the characteristic of being either perennial or temporary.

**Table 1:** Remote sensing data sources.

Data type	Source	Date	Temporal resolution	Spatial resolution
LST	MODIS/Terra^1^	2000–2008	8-days	1 km
NDVI	MODIS/Terra^1^	2000–2008	16-days	1 km
Land cover	MODIS/Terra^1^	2001–2004	Yearly	1 km
Rainfall	ADDS^2^	2000–2008	10-days	8 km
Altitude	DEM^3^	-	-	1 km
Freshwater bodies	HealthMapper^4^	-	-	Not known
Population counts	LandScan^5^	2008	-	1 km

1 Moderate Resolution Imaging Spectroradiometer (MODIS). Available at: https://lpdaac.usgs.gov/lpdaac/products/modis_products_table (accessed: 5 January 2009).

2 African Data Dissemination Service (ADDS). Available at: http://earlywarning.usgs.gov/adds/ (accessed: 5 January 2009).

3 Digital elevation model (DEM). Available at: http://eros.usgs.gov/ (accessed: 4 January 2009].

4 HealthMapper database. Available at: http://www.who.int/health_mapping/tools/healthmapper/en/index.html (accessed: 4 March 2009).

5 LandScan™ Global Population Database. Available at: http://www.ornl.gov/landscan/ (accessed: 20 January 2011).

Processing of the MODIS/Terra data was carried out using the ‘MODIS Reprojection Tool’ [20] and code implemented in Fortran 90 [21] to summarize the temporal changes by an overall yearly average based either on the mean (NDVI, day and night LST) or the mode (land cover). Furthermore, the land cover categories, as defined by the International Geosphere-Biosphere Programme, were re-grouped into six categories as follows: (i) sparsely vegetated; (ii) deciduous forest and savanna; (iii) evergreen forest; (iv) cropland; (v) urban; and (vi) wet areas. Rainfall estimates were processed via the software IDIRSI 32 [22]. Yearly averaged rainfall was calculated as summary measure. Distance calculations to the nearest freshwater body source were done in ArcMap version 9.2 of the Environmental Systems Research Institute (ESRI; Redlands, CA, USA) [23].

A classification scheme of West Africa into ecological zones was obtained using a demo version of the Earth Resources Data Analysis System Imagine 9.3 software [24]. The datasets were subjected to an unsupervised classification, via the ‘Iterative Self-Organizing Data Analysis Technique’ (ISODATA), to map areas of environmental clustering which were further summarized into five main classes based on between-class similarities. The resulting map matched existing classifications [25] and the classes can be interpreted as (i) desert/semi-desert; (ii) sahelian zone; (iii) savannah; (iv) forest; and (v) tropical rainforest.

Population count data obtained from LandScan for 2008 were converted to 5×5 km spatial resolution and adjusted to 2010 using country-specific average annual rates of change for 2005–2010 provided by the United Nations (UN) [26]. Estimates for the percentage of individuals aged ≤20 years among the total population per country were extracted from the U.S. Census Bureau International Database [27] for the year 2010. Population counts were linked to the percentage of children. The estimated number of infected individuals ≤20 years was calculated by combining a sample of the joint predictive posterior distribution of the disease prevalence predicted at pixel level with the population size of that age group within the pixel. The predictive posterior distribution of the number of infected individuals per country was estimated by summing up the pixel-samples and calculating summary statistics. The combined schistosomiasis prevalence (infection with *S. mansoni* or *S. haematobium* or both) was calculated on the assumption that the two infections are independent from each other, as *Schistosoma* spp. = *S. mansoni*+*S. haematobium*−(*S. mansoni* * *S. haematobium*).

Extraction of the remotely sensed data at the survey locations and at the prediction locations for the two databases was performed via a self written Fortran 90 code. The prediction surface for West Africa was built in ArcMap [23] with a spatial resolution of 0.05°×0.05° (approximately 5×5 km) resulting in approximately 220,000 pixels covering the study region. The data were displayed in ArcMap.

### Statistical analysis

For each *Schistosoma* species, bivariate logistic regressions were performed in STATA/IC 10.1 [28] in order to assess potential covariates in relation to the outcome (the number of infected individuals over the number of individuals screened per location). Continuous covariates were categorized into four groups based on quartiles to account for potential non-linearity in the outcome-predictor relationship on the logit. The Bayesian information criterion (BIC) was employed to detect whether linear or categorized covariates on the logits have smaller BIC and therefore predict the outcome more accurately. We used the following covariates in both linear and categorical scales: altitude, day LST, night LST, rainfall, NDVI, and distance to the nearest freshwater body. The type of freshwater body, ecological zone, and land cover were measured in categorical dimensions.

The study year was also included as linear and categorical covariate in order to account for possible temporal trends. The categories were defined on decades as follows: 1980–1989, 1990–1999, and from 2000 onwards. For *S. mansoni*, half of the data were from the 1980s (49.7%), 24.1% from the 1990s, whereas 26.2% were obtained in the new millennium. For *S. haematobium*, 37.8% of the data stem from the 1980s, 35.7% from the 1990s, and 26.5% from 2000 onwards.

Relevance of continuous or categorized covariates to predict the outcome was assessed based on p-values resulting from likelihood ratio tests (LRTs) at significance levels of 0.15. All significant covariates were included in the Bayesian analysis.

Bayesian geostatistical logistic regression models were fitted with location-specific random effects. Spatial correlation was modeled assuming that the random effects follow a multivariate normal distribution with variance-covariance matrix related to an exponential correlation function between any pair of locations. Model fit requires the inversion of this matrix. Due to the large number of survey locations in our datasets, parameter estimation becomes unfeasible. An approximation of the spatial process by a subset of 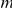 survey locations (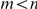) proposed by Banerjee *et al.* (2008) [29] and further developed by Gosoniu *et al.* (forthcoming) [30] and Rumisha *et al.* (forthcoming) [31] was implemented instead. We employed MCMC simulation to estimate the model parameters. Prevalence of infection at 220,000 locations was predicted for the most recent decade (from the year 2000 onwards) via Bayesian kriging using joint predictive posterior distributions [16]. Due to computational issues, we modeled the multivariate Gaussian spatial process separately for each country. The performance of the models was assessed using model validation via different approaches: mean predictive errors (ME), mean absolute predictive errors (MAE), discriminatory performance on a 50% prevalence cut-off, and Bayesian credible interval (BCI) comparisons [17]. Further details pertaining to the Bayesian geostatistical model, sub-sampling, and model validation approaches are given in the Appendix S1.

## Results

### Final datasets and preliminary statistics

**Figure 1:**
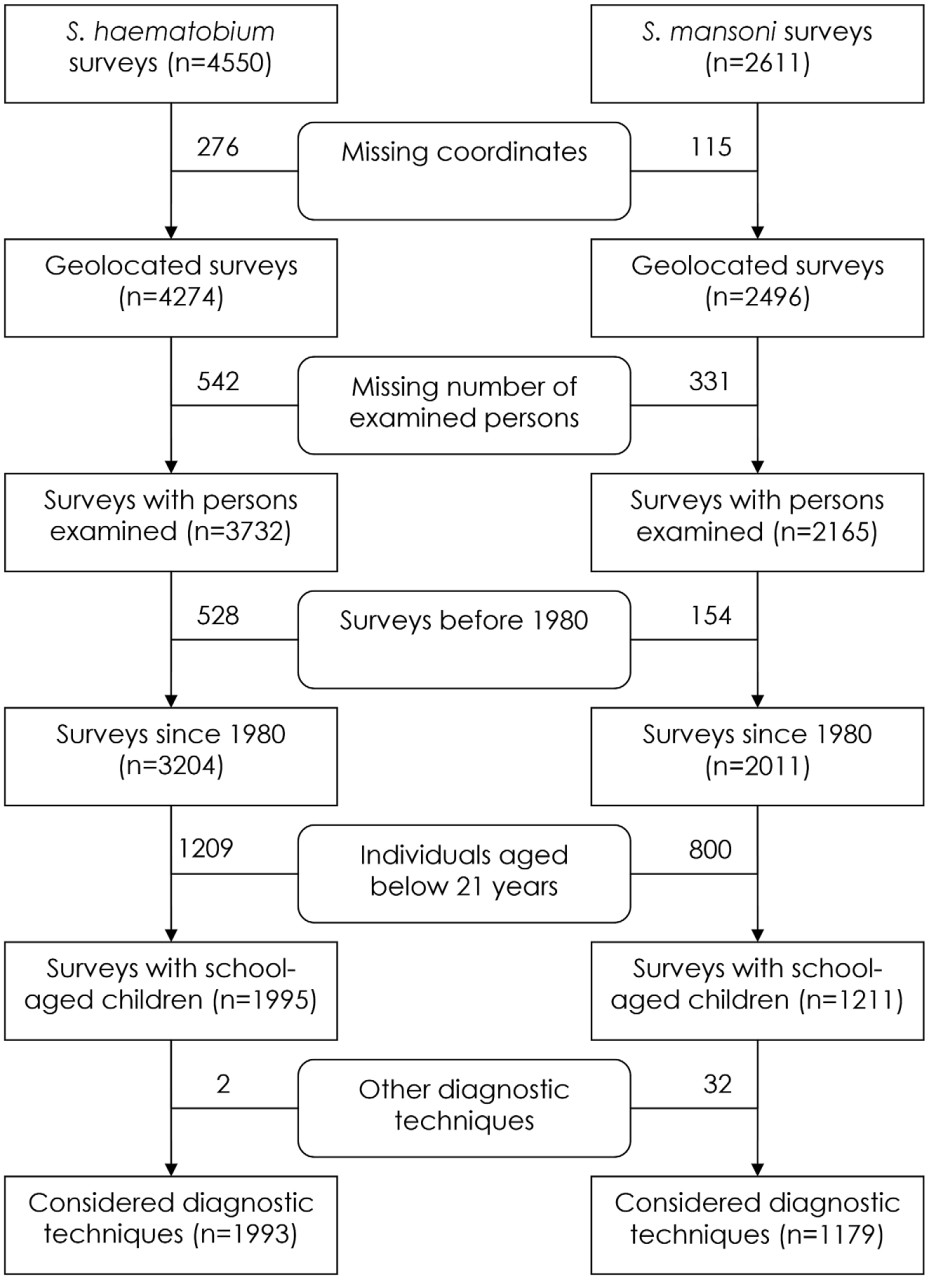
Study profile. Schematic overview of the study profiling process. The numbers in brackets in the acute-angled boxes represent the number of survey locations (which may not be unique) included in the current GNTD dataset, while the numbers outside the boxes represent the amount of survey dropped due to the reason given in the boxes with rounded corners.

**Figure 2:**
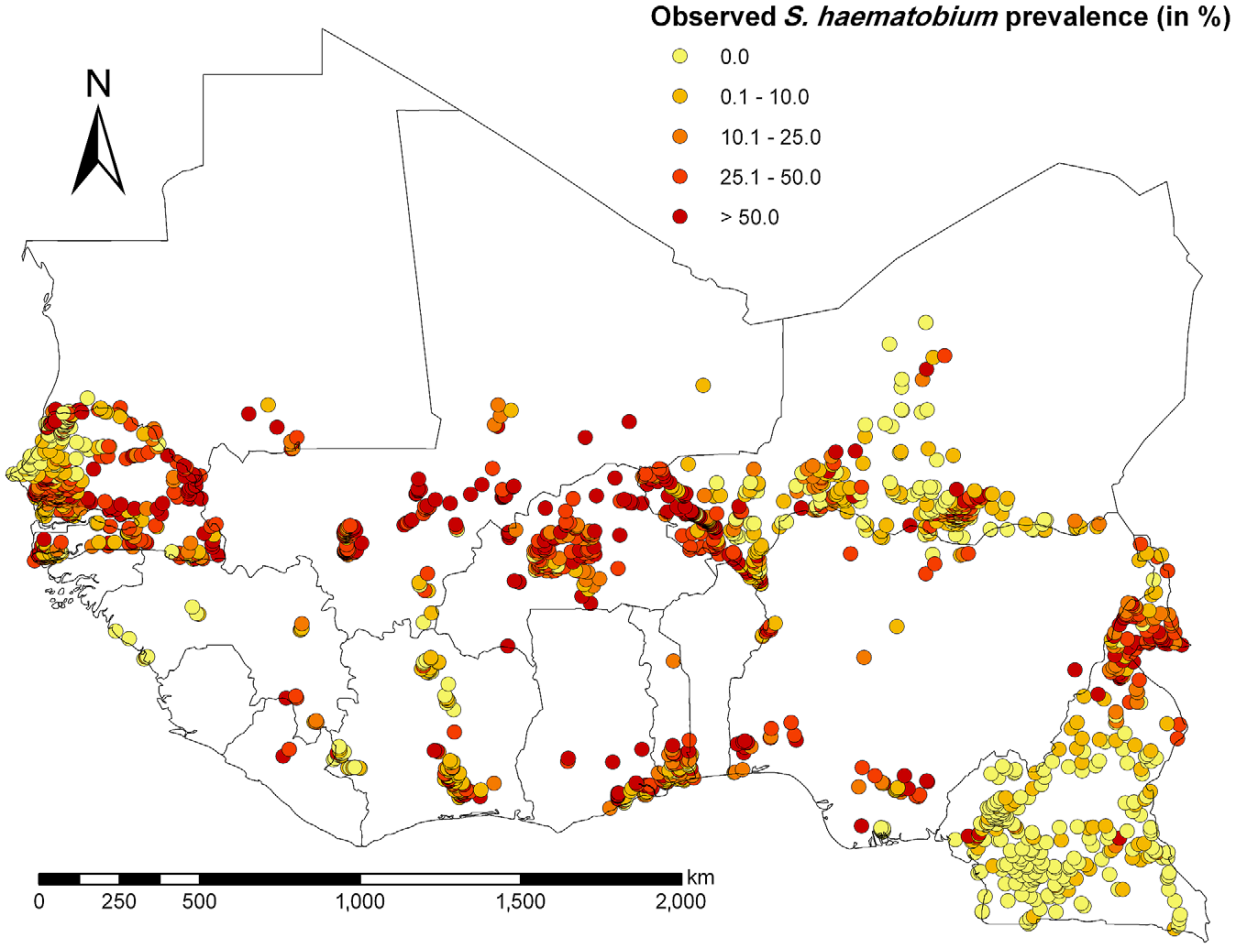
Observed *S. haematobium* prevalence in West Africa. Observed prevalence of *S. haematobium* among individuals aged ≤20 years across West Africa, including Cameroon.

**Figure 3:**
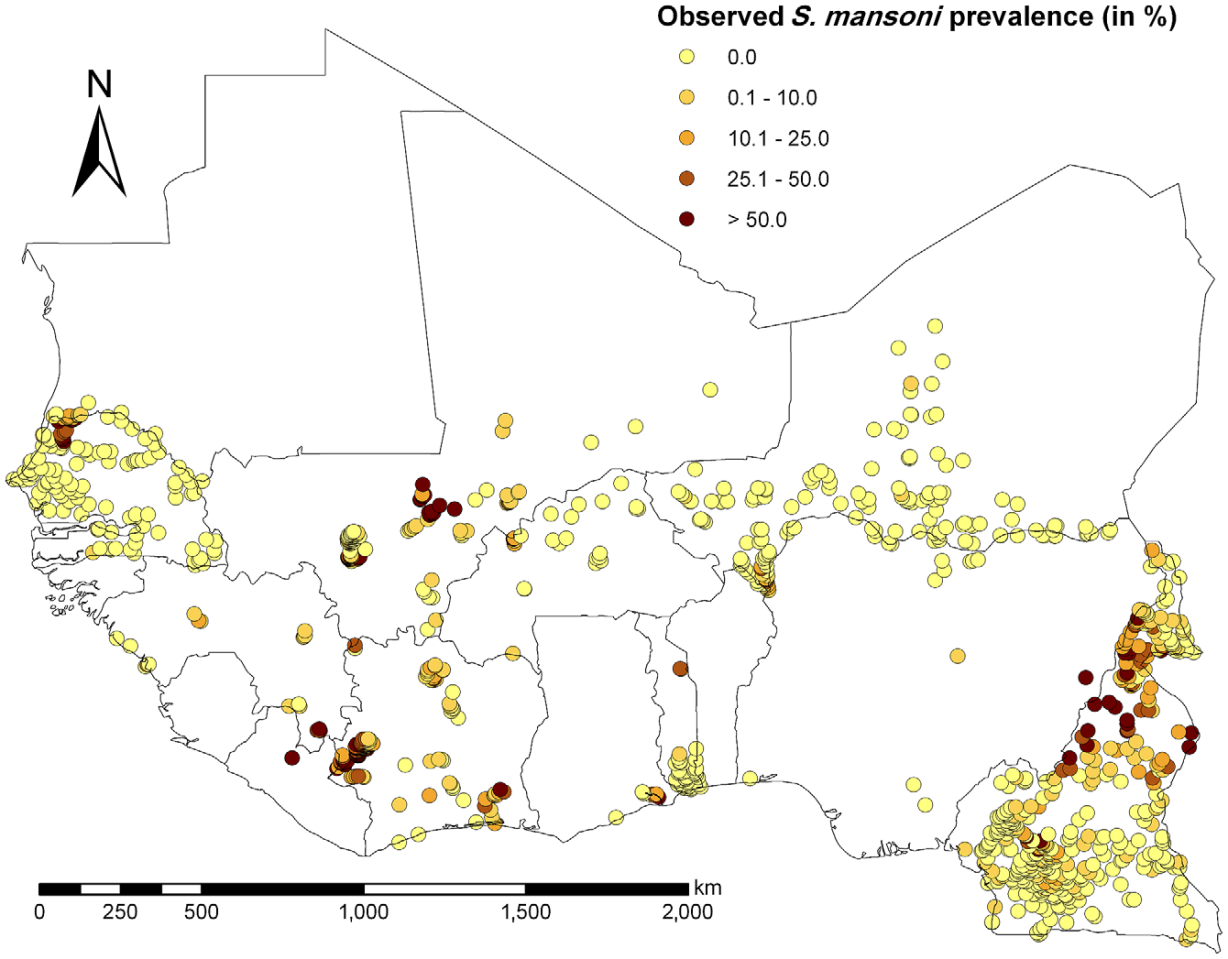
Observed *S. mansoni* prevalence in West Africa. Observed prevalence of *S. mansoni* among individuals aged ≤20 years across West Africa, including Cameroon.

A schematic overview of the study profile on obtaining prevalence data on schistosomiasis from the GNTD is given in Figure 1. The final datasets consisted of 1993 and 1179 survey locations for *S. haematobium* and *S. mansoni*, respectively, out of which 1722 and 1094 locations were unique. Observed prevalence of the survey locations ranged from 0% to 100% for each *Schistosoma* species with mean prevalence of 31.0% (median 15.0%, standard deviation (SD) 29.0%) for *S. haematobium*, and 17.7% (median 0.0%, SD 24.4%) for *S. mansoni*. The distribution and the prevalence level of the survey locations are shown in Figures 2 and 3 for *S. haematobium* and *S. mansoni*, respectively. An overview of the number of surveys with details given regarding sampling period, diagnostic technique, survey type, and mean prevalence, stratified by country, is given in Table 2.

**Table 2:** Overview on the survey data included in the analysis stratified by country.

	Locations		Survey year			Diagnostic technique*		Survey type	Preva-lence
	Total	Unique	1980s	1990s	2000+	UT	RS	School	Mean
***S. haematobium***									
Benin	5	5	0	5	0	5	0	5	18.2
Burkina Faso	123	119	92	8	23	35	88	117	46.4
Cameroon	349	342	335	4	10	18	0	342	22.2
Côte d'Ivoire	183	108	1	178	4	63	120	137	19.5
The Gambia	1	1	1	0	0	0	0	0	100.0
Ghana	47	47	22	8	17	47	0	36	38.5
Guinea	24	20	0	24	0	23	0	21	10.6
Guinea-Bissau	0	0	0	0	0	0	0	0	-
Liberia	3	2	3	0	0	3	0	0	51.3
Mali	139	130	83	23	33	137	0	33	45.4
Mauritania	27	25	8	11	8	27	0	19	34.8
Niger	544	442	104	304	136	473	0	455	32.7
Nigeria	86	71	36	21	29	80	1	48	38.3
Senegal	423	374	29	125	269	205	218	263	25.1
Sierra Leone	0	0	0	0	0	0	0	0	-
Togo	39	37	39	0	0	39	0	8	25.3
**Total**	**1993**	**1723**	**753**	**711**	**529**	**1155**	**427**	**1484**	**31.0**
***S. mansoni***						**KK**	**other**		
Benin	0	0	0	0	0	0	0	0	-
Burkina Faso	28	24	0	5	23	23	5	28	11.7
Cameroon	416	412	403	1	12	13	0	415	9.7
Côte d'Ivoire	201	157	12	118	71	200	0	141	33.3
The Gambia	0	0	0	0	0	0	0	0	-
Ghana	8	8	7	0	1	1	7	7	8.8
Guinea	22	20	0	22	0	22	0	20	12.7
Guinea-Bissau	0	0	0	0	0	0	0	0	-
Liberia	2	1	2	0	0	1	1	0	72.8
Mali	132	124	80	22	30	131	0	32	19.9
Mauritania	19	17	0	11	8	19	0	19	9.4
Niger	170	159	36	0	134	130	40	155	2.2
Nigeria	7	7	5	1	1	4	3	3	5.5
Senegal	133	126	0	104	29	132	0	27	18.2
Sierra Leone	0	0	0	0	0	0	0	0	-
Togo	41	39	41	0	0	38	3	8	4.4
**Total**	**1179**	**1094**	**586**	**284**	**309**	**714**	**59**	**855**	**17.7**

Details given on the number of surveys per survey year, diagnostic technique, survey type, and observed mean prevalence given per country and *Schistosoma* species.

*UT = urine test, RS = reagent strip, KK = Kato Katz thick smear.

**Figure 4:**
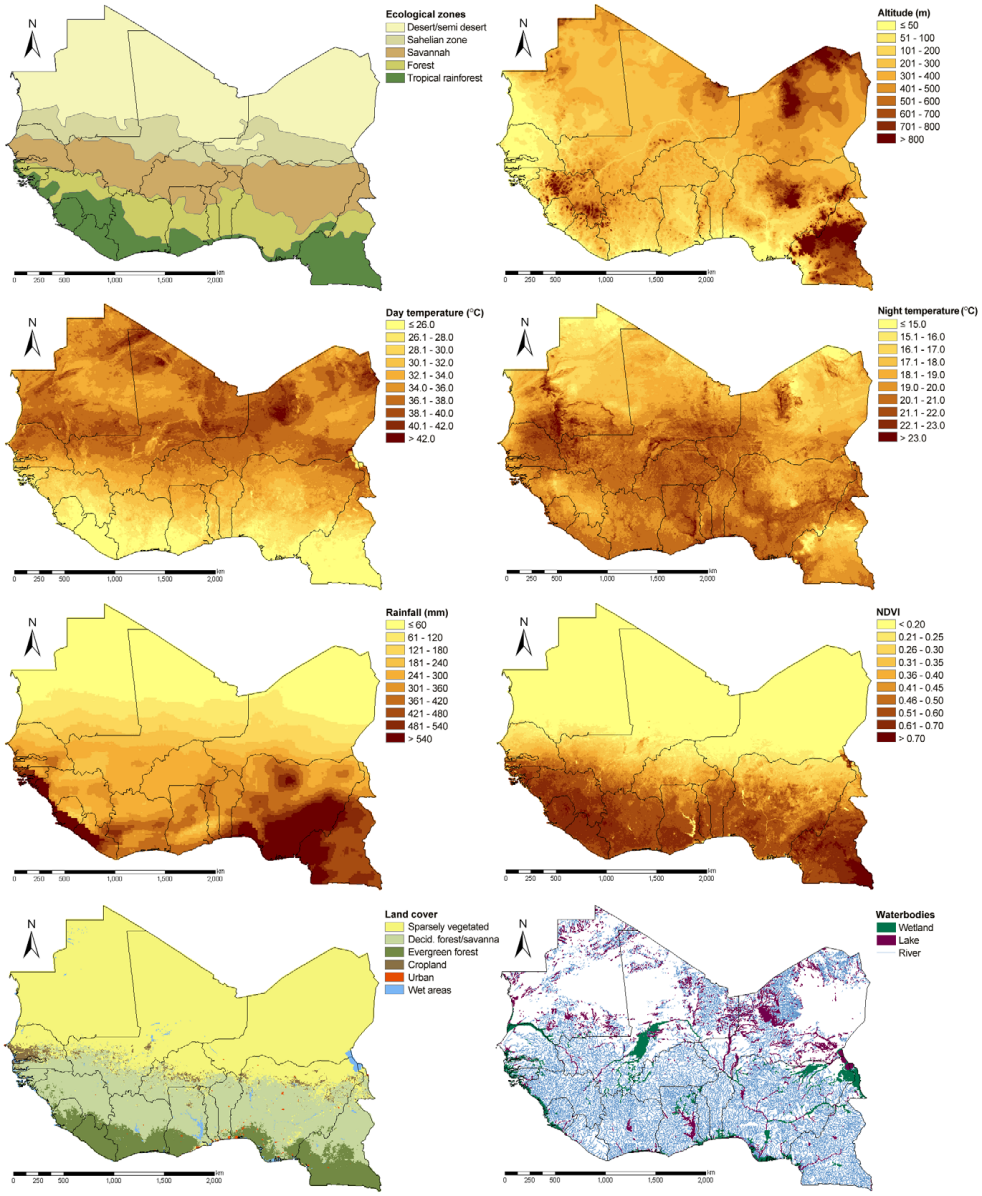
Remotely sensed covariates. Spatial distribution of remotely sensed covariates for West Africa, including Cameroon. Climatic covariates were summarized via yearly averages.

Spatial distributions of potential covariates influencing the distribution of schistosomiasis are presented in Figure 4. Bivariate logistic regressions of the continuous factors in relation to the disease outcomes showed that categorical variables predicted better based on BIC values than linear variables for both *Schistosoma* species (results not presented). Each potential covariate considered for the analyses had a p-value of <0.001 based on LRTs and was therefore included in the multivariate analyses. Backwards logistic regressions demonstrated the importance of the whole set of covariates for each species. The resulting odds ratios (ORs) of bivariate and multivariate non-spatial logistic regressions are summarized in Table 3 for *S. haematobium*, and Table 4 for *S. mansoni*. The only non-significant outcome-predictor relations in a multivariate framework for the former species were yearly averaged precipitation between 300 mm and 399 mm, and NDVI levels between 0.33 and 0.52. For the latter species, only altitude levels of at least 500 m above sea level and night LSTs between 20.0°C and 20.7°C were non-significant.

**Table 3:** Logistic regression parameter estimates for *S. haematobium*.

	Bivariate non-spatial	Multivariate non-spatial	Multivariate spatial
	OR (95% CI)	OR (95% CI)	OR (95% BCI)
**Decade**			
1980–1989	1.00	1.00	1.00
1990–1999	1.09 (1.07, 1.12)*	1.22 (1.18, 1.25)*	1.26 (1.22, 1.30)*
2000 onwards	1.16 (1.13, 1.19)*	1.26 (1.22, 1.29)*	1.14 (1.09, 1.20)*
**Ecological zone**			
Tropical rainforest	1.00	1.00	1.00
Forest	1.61 (1.56, 1.67)*	1.45 (1.40, 1.51)*	1.70 (1.63, 1.77)*
Savannah	2.05 (1.99, 2.12)*	2.33 (2.21, 2.46)*	1.28 (1.21, 1.36)*
Sahelian	1.97 (1.91, 2.03)*	2.05 (1.92, 2.19)*	1.01 (0.90, 1.14)
Desert/semi-desert	1.09 (0.99, 1.19)	1.35 (1.20, 1.52)*	0.57 (0.51, 0.65)*
**Altitude (m)**			
≤55	1.00	1.00	1.00
56–224	1.83 (1.78, 1.88)*	1.59 (1.55, 1.65)*	1.51 (1.45, 1.57)*
225–408	1.28 (1.25, 1.32)*	1.11 (1.07, 1.14)*	0.91 (0.86, 0.96)*
>408	0.81 (0.78, 0.83)*	1.32 (1.27, 1.37)*	0.93 (0.86, 1.00)
**Day LST (°C)**			
≤28.3	1.00	1.00	1.00
28.4–34.8	1.43 (1.39, 1.47)*	0.78 (0.75, 0.82)*	0.72 (0.68, 0.77)*
34.9–36.4	1.49 (1.45, 1.54)*	0.76 (0.71, 0.81)*	0.57 (0.53, 0.61)*
>36.4	1.19 (1.15, 1.22)*	0.63 (0.59, 0.67)*	0.49 (0.45, 0.53)*
**Night LST (°C)**			
≤19.2	1.00	1.00	1.00
19.3–20.4	2.15 (2.08, 2.23)*	1.86 (1.79, 1.93)*	1.70 (1.62, 1.79)*
20.5–21.1	2.84 (2.75, 2.94)*	2.52 (2.43, 2.62)*	1.99 (1.92, 2.05)*
>21.1	3.30 (3.20, 3.42)*	3.11 (2.99, 3.23)*	2.18 (2.12, 2.25)*
**Rainfall (mm)**			
0–249	1.00	1.00	1.00
250–299	1.45 (1.41, 1.49)*	1.13 (1.09, 1.18)*	1.16 (1.13, 1.21)*
300–399	1.12 (1.08, 1.15)*	0.95 (0.91, 1.00)	0.96 (0.92, 0.99)*
≥400	0.81 (0.78, 0.83)*	0.94 (0.89, 0.99)*	0.56 (0.51, 0.61)*
**NDVI**			
≤0.22	1.00	1.00	1.00
0.23–0.32	0.97 (0.94, 1.00)	1.05 (1.02, 1.09)*	0.96 (0.93, 0.99)*
0.33–0.52	0.93 (0.91, 0.96)*	1.05 (1.00, 1.10)	1.16 (1.13, 1.21)*
>0.52	0.67 (0.65, 0.69)*	0.91 (0.85, 0.97)*	1.20 (1.15, 1.25)*
**Land cover**			
Sparsely vegetated	1.00	1.00	1.00
Deciduous forest/savanna	0.72 (0.70, 0.74)*	0.72 (0.69, 0.75)*	0.78 (0.76, 0.80)*
Evergreen forest	0.75 (0.72, 0.77)*	1.13 (1.07, 1.20)*	1.36 (1.28, 1.42)*
Cropland	1.07 (1.04, 1.11)*	1.14 (1.10, 1.19)*	0.78 (0.75, 0.81)*
Urban	0.66 (0.64, 0.69)*	0.47 (0.45, 0.49)*	0.49 (0.46, 0.51)*
Wet areas	1.27 (1.18, 1.37)*	0.84 (0.77, 0.91)*	0.82 (0.75, 0.89)*
**Distance to closest freshwater body (km)**	0.95 (0.95, 0.95)*	0.98 (0.98, 0.99)*	0.98 (0.97, 0.98)*
**Type of closest water body**			
Perennial	1.00	1.00	1.00
Non-perennial	0.85 (0.83, 0.87)*	0.72 (0.70, 0.73)*	0.81 (0.78, 0.84)*
			**Mean (95% BCI)**
**Sigma^2^**	-	-	4.02 (3.37, 4.76)
**Range (km)**	-	-	398 (384, 412)

Logistic regression parameter estimates for *S. haematobium* summarized by odds ratios (OR), 95% confidence intervals (CI), and 95% Bayesian credible intervals (BCI).

*: Significant correlation based on 95% CI/BCI.

**Table 4:** Logistic regression parameter estimates for *S. mansoni*.

	Bivariate non-spatial	Multivariate non-spatial	Multivariate spatial
	OR (95% CI)	OR (95% CI)	OR (95% BCI)
**Decade**			
1980–1989	1.00	1.00	1.00
1990–1999	3.17 (3.03, 3.31)*	2.70 (2.55, 2.86)*	1.60 (1.46, 1.73)*
2000 onwards	1.82 (1.73, 1.91)*	1.36 (1.28, 1.44)*	1.14 (1.02, 1.28)*
**Ecological zone**			
Tropical rainforest	1.00	1.00	1.00
Forest	0.45 (0.42, 0.49)*	0.69 (0.61, 0.77)*	1.16 (1.01, 1.34)*
Savannah	0.40 (0.39, 0.42)*	0.78 (0.68, 0.89)*	0.20 (0.18, 0.23)*
Sahelian	0.82 (0.78, 0.85)*	3.22 (2.73, 3.80)*	0.07 (0.06, 0.08)*
Desert/semi-desert	0.01 (0.01, 0.02)*	0.05 (0.01, 0.20)*	0.01 (0.01, 0.01)*
**Altitude (m)**			
≤185	1.00	1.00	1.00
186–326	2.70 (2.57, 2.83)*	4.25 (3.98, 4.53)*	2.51 (2.32, 2.69)*
327–499	1.59 (1.52, 1.68)*	2.45 (2.29, 2.63)*	1.95 (1.70, 2.25)*
>499	0.98 (0.92, 1.04)	1.06 (0.97, 1.16)	1.80 (1.58, 2.05)*
**Day LST (°C)**			
≤25.0	1.00	1.00	1.00
25.1–31.2	0.83 (0.79, 0.87)*	1.45 (1.34, 1.56)*	1.34 (1.23, 1.45)*
31.3–35.6	0.78 (0.75, 0.82)*	1.90 (1.68, 2.15)*	2.05 (1.92, 2.18)*
>35.6	0.21 (0.19, 0.22)*	0.66 (0.57, 0.76)*	2.10 (1.88, 2.32)*
**Night LST (°C)**			
≤18.9	1.00	1.00	1.00
19.0–19.9	4.56 (4.30, 4.84)*	2.08 (1.94, 2.23)*	2.36 (2.18, 2.59)*
20.0–20.7	1.87 (1.76, 2.00)*	1.03 (0.95, 1.12)	0.97 (0.91, 1.03)
>20.7	0.92 (0.86, 0.99)*	0.47 (0.43, 0.51)*	0.46 (0.43, 0.50)*
**Rainfall (mm)**			
0–269	1.00	1.00	1.00
270–339	0.75 (0.71, 0.79)*	1.12 (1.03, 1.21)*	3.32 (2.89, 3.82)*
340–469	1.77 (1.69, 1.85)*	1.96 (1.77, 2.17)*	4.44 (3.97, 4.95)*
≥470	1.11 (1.05, 1.17)*	1.52 (1.36, 1.70)*	3.53 (3.16, 3.90)*
**NDVI**			
≤0.26	1.00	1.00	1.00
0.27–0.43	1.40 (1.33, 1.47)*	1.52 (1.39, 1.66)*	1.82 (1.62, 2.03)*
0.44–0.59	1.11 (1.05, 1.17)*	0.83 (0.73, 0.94)*	1.84 (1.52, 2.25)*
>0.59	2.97 (2.83, 3.12)*	1.45 (1.25, 1.67)*	0.94 (0.77, 1.15)
**Land cover**			
Sparsely vegetated	1.00	1.00	1.00
Deciduous forest/savanna	1.20 (1.14, 1.26)*	1.39 (1.28, 1.51)*	1.25 (1.17, 1.34)*
Evergreen forest	2.36 (2.26, 2.47)*	1.56 (1.40, 1.73)*	1.55 (1.45, 1.67)*
Cropland	1.46 (1.38, 1.55)*	1.51 (1.38, 1.66)*	0.82 (0.71, 0.94)*
Urban	1.41 (1.32, 1.50)*	1.27 (1.15, 1.41)*	1.72 (1.58, 1.88)*
Wet areas	0.47 (0.39, 0.57)*	0.62 (0.51, 0.76)*	0.60 (0.47, 0.77)*
**Distance to closest water body (km)**	0.92 (0.91, 0.92)*	0.91 (0.91, 0.92)*	0.94 (0.93, 0.94)*
**Type of closest water body**			
Perennial	1.00	1.00	1.00
Non-perennial	0.33 (0.32, 0.35)*	0.32 (0.31, 0.34)*	0.70 (0.64, 0.76)*
			**Mean (95% BCI)**
**Sigma^2^**	-	-	4.05 (3.37, 4.84)
**Range (km)**	-	-	387 (375, 402)

Logistic regression parameter estimates for *S. mansoni* summarized by odds ratios (OR), 95% confidence intervals (CI), and 95% Bayesian credible intervals (BCI).

*: Significant correlation based on 95% CI/BCI.

### Spatial modeling outcomes

Model parameter estimates for *S. haematobium* and *S. mansoni* are presented in Table 3 and Table 4, respectively. Introduction of spatial correlation led to changes in the significance of covariates and the direction of outcome-predictor relations compared to the corresponding non-spatial multivariate logistic regression models. For example, the influence of rainfall for *S. mansoni* became more important while the effect of the survey period and non-perennial freshwater bodies was reduced. The spatial range was estimated to be 398 km (95% BCI: 384–412 km) and 387 km (95% BCI: 375–402 km) for *S. haematobium* and *S. mansoni*, respectively. These estimates suggest strong spatial correlation for both species. The spatial variation was similar for the two species (4.02 for *S. haematobium vs.* 4.05 for *S. mansoni*).

### Schistosomiasis prevalence maps

**Figure 5:**
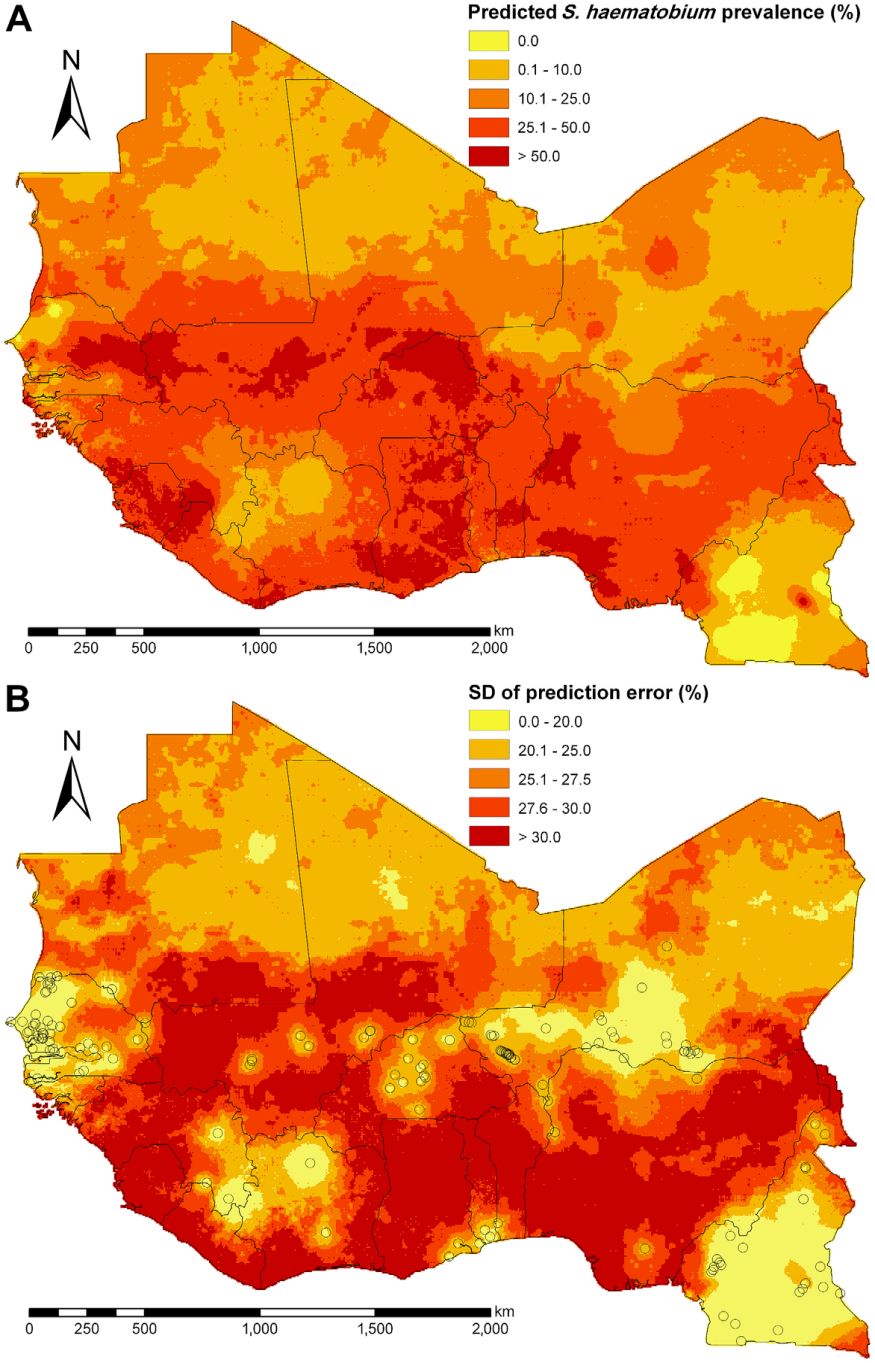
Predicted *S. haematobium* prevalence and standard deviation for West Africa. (A) Predicted median of prevalence for *S. haematobium* among individuals aged ≤20 years during the period of 2000–2009 based on Bayesian kriging, and (B) standard deviation (SD) of the prediction error with sub-sampled survey locations.

Figure 5A presents the prevalence map for *S. haematobium* based on the median of the predictions. Low-prevalence areas (predicted infection prevalence <10%) were primarily observed in the Sahara, Cameroon, north-west Côte d'Ivoire, and Senegal. Prevalence >50% are mainly spread along the Niger River, in Sierra Leone, east/central Senegal, and south Nigeria. The map of the SD of model predictions for this species (Figure 5B) demonstrates that small prediction errors were primarily found around the survey locations used for sub-sampling.

**Figure 6:**
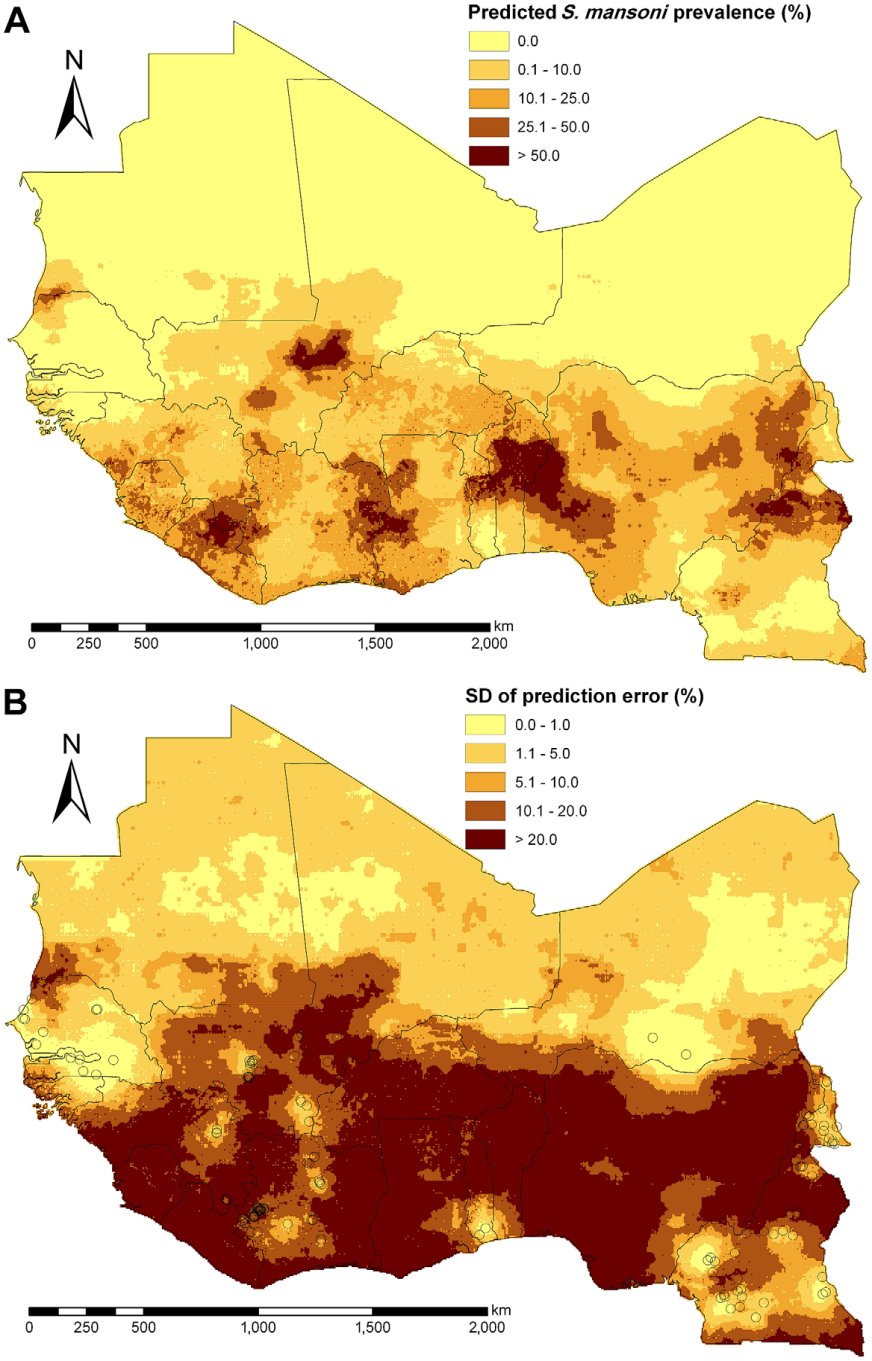
Predicted *S. mansoni* prevalence and standard deviation for West Africa. (A) Predicted median of prevalence for *S. mansoni* among individuals aged ≤20 years during the period of 2000–2009 based on Bayesian kriging, and (B) standard deviation (SD) of the prediction error with sub-sampled survey locations.

The median spatial *S. mansoni* prevalence map is shown in Figure 6A with the corresponding error presented in Figure 6B. High-prevalence areas (predicted prevalence >50%) were mainly found in north-east Liberia, east Côte d'Ivoire, west Ghana, north/central Benin, west Nigeria, north Cameroon, and central Mali in close proximity to Niger River. Very low prevalence areas (predicted prevalence <10%) were predominant in Senegal, The Gambia, Guinea-Bissau, Mauritania, and Niger. Furthermore, low prevalence areas were predicted for north Mali, south Togo, and parts of Cameroon. Areas of high prediction accuracy were found around the sub-sampled survey locations and in desert/semi-desert ecological zones.

### At-risk population estimates

Table 5 shows population-adjusted country prevalence estimates. For *S. haematobium*, prevalence estimates range between 17.6% (The Gambia) and 51.6% (Sierra Leone), whereas for *S. mansoni* they range between 0.5% (The Gambia) and 37.1% (Liberia). *S. haematobium* was found to be the predominant species throughout West Africa with a difference compared to *S. mansoni* of up to 30% in Burkina Faso and a minimum difference of about 4% in Liberia. Combined *Schistosoma* prevalence estimates, assuming independence of the occurrence of the two species, varied from 18.1% (The Gambia) to 58.3% (Liberia) with high numbers of infected individuals aged ≤20 years (more than 5 million) in Ghana and Nigeria. Lower numbers (<1 million) of infected individuals aged ≤20 years were found in The Gambia, Guinea-Bissau, Liberia, and Mauritania. The overall number of infected individuals aged ≤20 years in West Africa is 50.8 million.

**Table 5:** Prevalence and estimated number of infected children (0–20 years) per country.

	Population	*S. haematobium*		*S. mansoni*		*S. haematobium* & *S. mansoni*			
Country	Children (×10^6^)	Prevalence (%)	Infected (×10^6^)	Prevalence (%)	Infected (×10^6^)	Prevalence (%)	Infected (×10^6^)	Prevalence (%)^a^	Infected (×10^6^)^a^
		95% BCI	95% BCI	95% BCI	95% BCI	95% BCI	95% BCI		
Benin	4.620	38.8	1.792	20.3	0.940	46.0	2.124	35.5	1.950
		(18.0, 63.1)	(0.830, 2.914)	(5.9, 36.5)	(0.271, 1.687)	(22.1, 71.1)	(1.020, 3.282)		
Burkina Faso	9.434	45.4	4.282	15.3	1.446	50.2	4.738	60.0	6.240
		(32.3, 59.4)	(3.043, 5.606)	(4.5, 38.2)	(0.427, 3.604)	(34.7, 67.8)	(3.274, 6.400)		
Cameroon	10.300	20.4	2.099	9.2	0.952	25.9	2.668	26.5	3.020
		(13.5, 29.0)	(1.389, 2.986)	(6.9, 12.5)	(0.715, 1.289)	(18.8, 34.7)	(1.934, 3.573)		
Côte d'Ivoire	11.000	31.5	3.229	22.1	2.262	41.8	4.286	40.0	5.600
		(16.4, 50.9)	(1.677, 5.213)	(12.6, 35.5)	(1.293, 3.642)	(25.4, 60.8)	(2.605, 6.235)		
The Gambia	4.872	17.6	0.168	0.5	0.005	18.1	0.173	37.5	0.330
		(9.3, 36.9)	(0.088, 0.352)	(0.0, 5.5)	(0.000, 0.053)	(9.3, 38.7)	(0.089, 0.369)		
Ghana	0.822	46.1	5.077	24.2	2.659	53.7	5.912	72.5	12.400
		(26.5, 67.2)	(2.918, 7.396)	(9.8, 49.5)	(1.081, 5.452)	(31.0, 76.0)	(3.408, 8.365)		
Guinea	10.300	37.4	1.824	20.5	0.999	46.4	2.259	25.8	1.700
		(18.8, 57.0)	(0.914, 2.776)	(9.3, 35.9)	(0.455, 1.749)	(25.8, 66.0)	(1.255, 3.214)		
Guinea-Bissau	0.953	24.7	0.203	2.9	0.024	26.5	0.218	30.0	0.330
		(6.7, 59.6)	(0.055, 0.490)	(0.2, 21.3)	(0.002, 0.175)	(7.0, 63.0)	(0.057, 0.518)		
Liberia	1.585	41.5	0.658	37.1	0.588	58.3	0.924	30.0	0.648
		(14.7, 69.5)	(0.233, 1.102)	(14.1, 66.3)	(0.223, 1.051)	(24.6, 84.4)	(0.390, 1.338)		
Mali	4.430	45.1	1.997	19.1	0.845	51.7	2.291	60.0	5.880
		(27.9, 63.2)	(1.237, 2.801)	(13.0, 27.2)	(0.573, 1.204)	(35.5, 67.7)	(1.572, 3.000)		
Mauritania	0.944	31.7	0.299	5.8	0.055	35.2	0.333	27.4	0.630
		(19.0, 46.6)	(0.180, 0.44)	(2.7, 10.8)	(0.026, 0.101)	(22.0, 51.1)	(0.208, 0.483)		
Niger	5.160	25.6	1.321	3.5	0.179	27.1	1.397	26.7	2.400
		(19.4, 33.2)	(1.001, 1.712)	(0.6, 12.1)	(0.031, 0.625)	(19.9, 35.7)	(1.028, 1.841)		
Nigeria	39.900	39.4	15.741	23.2	9.257	47.0	18.754	25.2	25.830
		(24.7, 55.7)	(9.866, 2.253)	(11.8, 38.0)	(4.717, 5.175)	(30.0, 63.9)	(11.976, 25.505)		
Senegal	6.358	21.0	1.338	2.9	0.183	23.0	1.464	15.3	1.300
		(16.7, 24.9)	(1.062, 1.581)	(1.5, 5.9)	(0.094, 0.372)	(18.1, 27.6)	(1.151, 1.755)		
Sierra Leone	3.476	51.6	1.792	24.5	0.853	57.5	1.999	67.6	2.500
		(15.4, 84.7)	(0.535, 2.944)	(4.4, 59.8)	(0.153, 2.080)	(17.6, 89.6)	(0.612, 3.113)		
Togo	2.985	36.9	1.102	14.0	0.419	41.9	1.251	25.1	1.030
		(18.1, 58.5)	(0.540, 1.745)	(3.6, 31.4)	(0.107, 0.938)	(21.0, 62.6)	(0.628, 1.869)		

Median prevalence and estimated number of infected individuals (aged ≤20 years) per country (predicted for the period 2000–2009) based on 2010 population estimates with 95% Bayesian credible interval (BCI).

a Estimated country prevalence and number of infected individuals with schistosomiasis over all age groups in 1995 as presented by Chitsulo *et al.* (2000) [4].

### Model validation results

Model validation based on 80% of the survey locations resulted in MEs of −1.7 for *S. haematobium* and 0.0 for *S. mansoni*, and respective MAEs of 19.5 and 7.3. The percentage of test locations correctly predicted by 95% BCIs was 72.9% for *S. haematobium*, and 72.5% for *S. mansoni*. ME and MAE comparisons between spatial and exchangeable random effect models showed that spatial models result in better predictive ability (*S. haematobium*: ME = 3.8, MAE = 27.7; *S. mansoni*: ME = −0.8, MAE = 14.9).

Discriminatory performance based on a 50% prevalence cut-off showed that the models correctly predicted 93.2% and 76.9% of the validation locations for *S. mansoni* and *S. haematobium*, respectively. False-high predictions were obtained for 5.5% (*S. mansoni*) and 18.8% (*S. haematobium*) of the test locations.

## Discussion

To our knowledge, we provide the first model-based prevalence maps for both *S. haematobium* and *S. mansoni* for individuals aged ≤20 years in West Africa, including Cameroon. We used a readily available open-access database consisting of a large number of historical and contemporary geolocated and standardized survey data [15], coupled with Bayesian-based geostatistical tools. Standard geostatistical methods are not able to handle large numbers of survey locations due to computational problems. Therefore, for the first time, an approximation of the spatial process was implemented in *Schistosoma* prevalence modeling.

In comparison to existing prevalence estimates, major shortcomings of previous studies have been addressed, and hence our prevalence maps show a higher spatial resolution and we believe that they are more accurate than heretofore. This claim is justified as follows. First, our estimates are based on the GNTD database that has gone live in July 2010, developed as part of the EU-funded CONTRAST project. As of February 2010, the GNTD contained more than 4500 and 2600 unique entries in West Africa for *S. haematobium* and *S. mansoni*, respectively. Second, data-tailored statistical methods based on Bayesian geostatistical modeling were used in order to incorporate spatial correlation between survey locations and to obtain more accurate estimates of the uncertainty of the predictions. Third, climatic and environmental covariates were employed in the models to evaluate the effect on the disease outcomes. The climatic and environmental factors were obtained at high spatial resolution to be able to predict small hotspots of risk, which could arise due to the focal distribution of schistosomiasis, which is an important epidemiological feature of the disease [32]. An existing *S. haematobium* prevalence map for three West African countries (i.e., Burkina Faso, Mali, and Niger) using Bayesian geostatistical modeling was previously presented by Clements *et al.* (2008) [33] based on data from 2004–2006. However, this map does not show the actual level of schistosomiasis prevalence but rather probabilities that the predicted prevalence is above a pre-defined cut-off, arbitrarily set at 50%. This cut-off has been proposed by the World Health Organization (WHO) [1] to distinguish between low and high risk areas, and hence such maps are useful to detect areas where preventive chemotherapy might be warranted on an annual basis. However, the maps do not provide detailed information for lower risk areas or the number of infected individuals and they cannot be used for monitoring and evaluation purposes following interventions. A more recent publication by Clements *et al.* (2009) [9] presented a *S. haematobium* prevalence map for the same three West African countries. This map shows similar patterns to our map with the exception of north Burkina Faso. In this area, Clements and colleagues predicted prevalence levels of 10–20% for high and low egg-intensities, while our estimates suggest much higher prevalence (>50%). These discrepancies are most likely due to differences in the underlying survey data. The Clements *et al.* data were only partially included in the GNTD database as we could not access them fully.

The estimated spatial correlation for both *Schistosoma* species was very strong with spatial ranges of approximately 400 km. Previously reported spatial ranges in parts of West Africa vary between 7.5 km [19] and approximately 180 km [33]. However, these estimates were based on recent surveys, and hence influenced by recently established control programs. Interventions are likely to reduce the predictive power of environmental and climatic factors on the distribution of schistosomiasis and, thus, reduce spatial correlation. Similar effects were found for malaria, where historic data showed stronger spatial correlation [34] than recent surveys [35], [36].

We overlaid population data adjusted to 2010 on the predicted prevalence surfaces for the two *Schistosoma* species in order to obtain country-specific estimates of the number of infected individuals aged ≤20 years. Previous country estimates, for instance those presented by Chitsulo *et al.* (2000) [4], Steinmann *et al.* (2006) [2], or Utzinger *et al.* (2009) [5], are interpolations of limited observations for a whole country, and hence lack empirical modeling. Chitsulo and colleagues reported a higher number of infected people for West Africa (71.8 million) compared to our estimate (50.8 million). Of note, the Chitsulo *et al.* estimates are based on the whole population, while our new estimates concern the age group ≤20 years. Moreover, the Chitsulo *et al.* estimates pertain to mid-1990s population estimates, compared to our adjusted estimates for the year 2010. In countries like Cameroon, The Gambia, Ghana, and Liberia, characterized by high rural-to-urban migration in the last decade, the Chitsulo *et al.* prevalence estimates should be treated with care due to rapid urbanization. Our study revealed that the combined prevalence of *S. haematobium* and *S. mansoni* in The Gambia, for example, is two-fold lower than previously reported by Chitsulo *et al.* (18.1% *vs.* 37.5%). However, in Benin, Guinea, Liberia, Nigeria, and Togo, we found prevalence estimates that are more than 10 percentage points higher than the previous estimates. On the one hand, differences might be related to sparse data, for example, in Benin, The Gambia, Guinea, Guinea-Bissau, Liberia, Mauritania, Nigeria, and Sierra Leone. Previous estimates failed to take into account model-based predictions on the basis of climate, environment and disease data. Since we modeled disease prevalence on individuals aged ≤20 years (highest risk groups), the prevalence estimates correspond to the former risk group. Therefore they are likely to overestimate the prevalence in the whole population.

We estimated the country-specific overall schistosomiasis prevalence by assuming independence between the occurrence of *S. haematobium* and *S. mansoni* in each area. However, it is conceivable that simultaneous infections with both species is more frequent than expected by chance in areas where the species co-exist as infection pathways are similar and highly behavioral related. Hence, the combined prevalence estimates potentially underestimate the true schistosomiasis situation in West Africa. A modeling approach via joint spatial random effects [37] could assess the effect of potential dependence between the species, but would increase the number of spatial parameters and is therefore computationally challenging.

We might also underestimate schistosomiasis prevalence in Cameroon, Mali, and Nigeria because of the presence of *S. intercalatum* [4]. We did not include this species in the analysis since the GNTD database currently only contains 17 survey locations outside Cameroon. However, it is assumed that *S. intercalatum* has a low prevalence [4] and there are signs that this species is further declining in importance [38].

Model validation has shown that the *S. haematobium* predictions seem to overestimate the actual prevalence, while the *S. mansoni* model revealed no tendency to over- or underestimate the overall prevalence. The MAE for the *S. haematobium* model is nearly three times larger than the one for *S. mansoni*. This is expected because the mean prevalence for *S. haematobium* was about double than that for *S. mansoni*. Our models correctly predict about 72% of the survey locations when considering 95% BCIs. We are encouraged by these results, since perfect predictions are rather unlikely in reality due to the complexity of disease transmission.

However, our models are based on assumptions, which could influence model performance. We assumed that the diagnostic techniques employed have similar ability to detect an infection, but different diagnostic techniques show differences in sensitivity and specificity, which also depends on the overall prevalence and infection intensity [39]. This might have led to an underestimation of prevalence due to the imperfect sensitivity of direct diagnostic techniques [39]. Additional model parameters accounting for the performance of the different diagnostic techniques could be incorporated in the models. However in the absence of detailed information regarding sampling effort, assumptions would be required which may be debatable and introduce additional biases. We are currently examining the effect of different approaches on addressing this issue on the model-based predictions.

We did not adjust the outcome according to age and sex even though the age groups differ and especially school surveys are likely to include more boys than girls due to prevailing cultural issues in many parts of West Africa. Therefore, our results are likely to be biased and potentially overestimate schistosome prevalence. However, many publications do not present stratified results by these subgroups. Age-adjustment models are feasible but difficult to implement because age-prevalence curves have to be fitted for different transmission settings [40]. Furthermore, disease data are often reported at wide age ranges (i.e., school-aged children) and individuals might not be well distributed within the range introducing bias even though an age-prevalence model is taken into account.

Surveys are typically conducted in endemic areas leading to high observed prevalence levels. This could result in an overestimation of prevalence in the present analysis. However, in the data we analyzed, 45% of the locations for *S. haematobium* and 73% for *S. mansoni* had an observed prevalence levels below 10%. We therefore assume that a location selection bias is unlikely. Another concern is the large amount of zero outcomes (i.e., none of the study participants found to be infected) especially for *S. mansoni* (*S. mansoni*: 54.1%; *S. haematobium*: 20.1%). To overcome this issue, zero-inflated models need to be incorporated, which modify the likelihood function and add an additional model parameter capturing the over-dispersion arising by the zeros [41].

The models presented in this manuscript did only include spatial random errors, and hence we ignored potential measurement errors. Inclusion of location-specific non-spatial error terms might have improved model predictions. However, location-specific non-spatial error terms would have doubled the number of error terms leading to highly parameterized models.

We further assumed isotropic stationary models. Non-stationary models imply that the spatial random effect is varying from one region to another and is not stable throughout the study area [35]. This assumption has been confirmed by semi-variogram comparisons showing that the estimated spatial range parameters for *S. mansoni* differ between eco-zones. However, semi-variogram analyses did not indicate non-stationarity in the spatial distribution of *S. haematobium*. Isotropic models assume that the spatial correlation is the same within the same distance irrespective of direction [42]. This assumption might not be valid since intermediate host snails spread along rivers and lakeshores and, therefore, introduce correlation attributed to directions.

The choice and size of sub-sampled locations required to adequately approximate the spatial Gaussian process is a research area on its own in spatial statistics. Many different approaches are available to optimize selection. We implemented a method based on semi-variogram comparisons. This selection is aiming to preserve the spatial surface of the original dataset. However, it might fail to identify a sub-sample, which minimizes the prediction error. The spatially averaged predictive variance (SAPV) method proposed by Finley is trying to optimize the variance in the predictions, but implementation is computationally highly demanding [43].

Time-dependent covariates, such as the climatic factors, might have changed between the 1980s and the 2000s. However, our geographical covariates were solely based on recent remote sensing data (from 2000 onwards), because historical remote sensing data are, to our knowledge, not freely available at high spatial and temporal resolution. The long run averages of the recent data enable us to maintain high spatial resolution although they cannot capture variation in the observed outcome due to unusual climatic conditions or climate change that might have occurred since the 1980s and 1990s.

Preliminary residual analyses suggest that there is only weak temporal correlation in the data. We therefore only modeled a spatial rather than a spatio-temporal process. This led to a more parsimonious model and facilitated model fit. Nevertheless, we incorporated temporal trends in the prevalence estimation by including the survey year as covariate. Both *Schistosoma* species showed that the predicted prevalence was highest during the 1990s. This increase might be explained by water resources development and management activities (e.g., the construction of dams and irrigation systems), political unrests and civil restructuring. Water resources development and management projects might have improved the suitability of the environment for snail intermediate hosts that might have spread into previously snail-free zones together with the parasites. Since the beginning of the new millennium, a number of large-scale preventive chemotherapy programs are underway in parts of West Africa and it will be important to monitor how the prevalence of schistosomiasis changes in space and over time. The effectiveness of control interventions may vary across areas but, to our knowledge, a comprehensive database compiling this information with high spatio-temporal resolution has yet to be established.

Concluding, our country-specific *Schistosoma* prevalence estimates and numbers of individuals aged ≤20 years infected with either *S. mansoni*, or *S. haematobium*, or both species concurrently presented here are useful tools for disease control managers and other stakeholders to support decision-making on interventions. Our maps can also serve as a benchmark to monitor the impact of control interventions and for long-term evaluation on transmission dynamics. Model-based estimates in areas with scarce data and high uncertainty could be improved by additional surveys to enhance our knowledge on the distribution of schistosomiasis and disease burden. We plan to further expand this work to other regions and address the issues of non-stationarity, diagnostic sensitivity, and age-heterogeneity across surveys. Finally, we will test the assumption of independence between the *Schistosoma* species to improve accuracy of the joint prevalence estimates.

## Supporting Information

Alternative Language Abstract S1 **Geostatistische modellbasierte Abschätzungen zur Häufigkeit von Schistosomiasis in Westafrika für Personen im Alter von maximal 20 Jahren - Translation of abstract into German by Nadine Schur.**
(PDF)

Appendix S1 **Supporting information on geostatistical model formulation, spatial process approximation and model validation.**
(PDF)

Checklist S1 **STROBE checklist.**
(PDF)
