# Supplementary material for: Geostatistical Model-Based Estimates of Schistosomiasis Prevalence among Individuals Aged ≤20 Years in West Africa
Source: PLoS Negl Trop Dis. 2011 Jun 14;5(6):e1194. doi: 10.1371/journal.pntd.0001194 (PMC3114755; doi:10.1371/journal.pntd.0001194)
Supplement: Alternative Language Abstract S1 — Geostatistische modellbasierte Abschätzungen zur Häufigkeit von Schistosomiasis in Westafrika für Personen im Alter von maximal 20 Jahren - Translation of abstract into German by Nadine Schur. (PDF) [file pntd.0001194.s001.pdf]

Geostatistische modellbasierte Abschätzungen zur Häufigkeit von Schistosomiasis in Westafrika für Personen im Alter von maximal 20 Jahren

Zusammenfassung

Hintergrund: Schistosomiasis ist eine wasserbasierte Krankheit die vermutlich mehr als 200 Millionen Menschen betrifft, v.a. in Afrika wo geschätzte 97% der Infektionen vorkommen. Allerdings basieren diese Statistiken zum Grossteil auf bevölkerungsbereinigten Daten, welche von Utroska und Mitarbeitern vor mehr als 20 Jahren veröffentlicht wurden. Diese ursprünglichen Schätzungen sind vermutlich veraltet aufgrund von groß angelegten Kontrollprogrammen, verbesserter Hygiene und dem Ausbau von Wasserressourcen Projekten. Für die Planung, Durchführung und Auswertung von Kontrollmaßnahmen ist es indes wichtig, verlässliche Angaben zur räumlichen Verteilung der Erkrankung zu haben.

Methodik: Wir analysierten Studiendaten, extrahiert aus einer neuen, globalen und frei zugänglichen Datenbank über vernachlässigte tropische Krankheiten, um (i) empirische Kartierungen von *Schistosoma mansoni* und *S. haematobium* für Personen im Alter von maximal 20 Jahren in Westafrika (und Kamerun) zu erhalten, und (ii) länderspezifische Häufigkeiten abzuschätzen. Dazu verwendeten wir Bayes'sche geostatistische Modelle auf Basis von Umweltfaktoren um mögliche Ballungen aufgrund gemeinsamer räumlicher Einflüsse berücksichtigen zu können. Vorhersagen an Orten ohne bisherige Studien wurden mittels Kriging-Verfahren durchgeführt.

Wichtigste Ergebnisse: Unsere Modelle zeigen, dass derzeit etwa 50,8 Millionen Westafrikaner im Alter von maximal 20 Jahren entweder mit *S. mansoni*, *S. haematobium*, oder mit beiden Arten gleichzeitig, infiziert sind. Länderspezifische Prävalenz-Schätzungen lagen zwischen 0,5% (Gambia) und 37,1% (Liberia) für *S. mansoni* und zwischen 17,6% (Gambia) und 51,6% (Sierra Leone) für *S. haematobium*. Die gemeinsame Prävelenz für beide Arten in Gambia lag unterer der Hälfte der bisherigen Annahme, wohingegen wir fast doppelt so hohe Werte als zuvor in Liberia ermittelt haben (58,3% *vs.* 30,0%). Allerdings überschätzen unsere Vorhersagen vermutlich die tatsächliche Lage, da die Modellierung ausschließlich auf Kindern und Jugendlichen im Alter von bis zu 20 Jahren beruht, welche die höchste Risikogruppe für Schistosomiasis Infektionen darstellen.

Schlussfolgerung/Bedeutung: Wir präsentieren die ersten empirischen Schätzungen mit hoher räumlicher Auflösung zur Häufigkeit von *S. mansoni* und *S. haematobium* für das gesamte westafrikanische Gebiet. Unsere Risikokarten erlauben es räumliche Prioritäten bei der Umsetzung von Interventionen zu setzen. Zudem sind die Karten nützliche Werkzeuge zur Überwachung und Bewertung von Kontrollprogrammen der Erkrankung.

Übersetzung: Nadine Schur
