# Supplementary material for: Geostatistical Model-Based Estimates of Schistosomiasis Prevalence among Individuals Aged ≤20 Years in West Africa
Source: PLoS Negl Trop Dis. 2011 Jun 14;5(6):e1194. doi: 10.1371/journal.pntd.0001194 (PMC3114755; doi:10.1371/journal.pntd.0001194)
Supplement: Appendix S1 — Supporting information on geostatistical model formulation, spatial process approximation and model validation. (PDF) [file pntd.0001194.s002.pdf]

Appendix

Geostatistical modelling

Let Y_i and N_i be the number of infected and screened individuals at location i ($i = 1, \ldots, n$) and p_i the probability of infection. We assume that Y_i arises from a Binomial distribution, i.e., $Y_i \sim Bin(p_i, N_i)$. The influence of covariates $\underline{X}_i$ and location-specific spatial random effects w_i are modelled on the logit, as $\log it(p_i) = \underline{X}_i^T \underline{\beta} + w_i$, where $\underline{\beta}$ is the vector of regression coefficients. Unobserved spatial variation is introduced on w_i by assuming that $\underline{w} = (w_1, \ldots, w_n)^T$ follows a latent stationary Gaussian process over the study region, $\underline{w} \sim MVN(\underline{0}, \Sigma)$. Σ is a matrix with elements Σ_{ij} accounting for the covariance between any pair of locations i and j. Assuming an isotropic exponential correlation function, the matrix elements are defined by $\Sigma_{ij} = \sigma^2 \exp(-\rho d_{ij})$ with spatial variance σ^2, rate of correlation decay ρ and the distance between locations d_{ij}. The data are spread over large areas and Euclidean distances are not appropriate any longer, since they are unable to account for the curvature of the surface of the Earth. Therefore, the great-circle distance was used [44]. The minimum distance for which the spatial correlation is less than 5% is referred to as range and can be calculated by $3/\rho$ in the exponential correlation function setting.

A Bayesian model formulation requires the specification of prior distributions of all model parameters. For the regression coefficients $\underline{\beta}$, we assumed Normal prior distributions with mean 0 and large variance. For the spatial parameters σ^2 and ρ, we chose non-informative inverse Gamma and Gamma distributions, respectively.

The model was fitted using Markov chain Monte Carlo (MCMC) simulation implemented in Fortran 90 [21] code written by the investigators using the standard numerical libraries [45]. The code was run with two chains and a burn-in of 5000 iterations. Starting values for the chains were based on non-spatial model estimates from STATA/IC 10.1 [27] and semi-variogram estimates for the spatial model parameters. Convergence was assessed by inspection of ergodic averages of selected model parameters during the sampling period of 50,000 iterations. The models converged after approximately 30,000 iterations. Samples of 500 iterations per chain were saved for each model.

Predictive posterior distributions at the 220,000 prediction locations were estimated via Bayesian kriging [16] implemented in Fortran 90 [21] using the standard numerical libraries [45]. Our predictions are based on the period from 2000 onwards.

Spatial process approximation

Depending on the number of survey location, parameter estimation can be very slow or infeasible (computational costs are in the order of n^3), because the variance-covariance matrix of the spatial process $\Sigma_{n \times n}$ needs to be inverted at every iteration during the fitting and kriging process. Each of the processed datasets for *S. mansoni* and *S. haematobium* in West Africa includes more than 1000 unique survey locations, therefore it is not possible to include the full matrix to estimate spatial correlation between locations. We overcame the computational burden by an approximation of the spatial process via a subset of m survey locations ($m < n$) [29].

The subset was selected via balanced sampling [46] with a modified inclusion probability based on the variability of the outcome [30,31]. A grid of 15 equally sized tiles ($A_i, i = 1, \ldots, 15$)

was created over the study area and each survey location was allocated to the tile surrounding it. The within-tile variability σ_a^2 and total variability σ_A^2 were assessed and the inclusion probability of a location within a specific tile a was calculated by σ_a^2/σ_A^2. Sampling of the locations based on the inclusion probability and upon the selected covariates was performed in R 2.10.0 [47] via the 'samplecube' function of the 'sampling' library.

A semi-variogram analysis was performed to identify the minimum size of the sub-sample still preserving the spatial correlation surface of the original datasets of the two *Schistosoma* species. The location subset of choice was used in model fit as a proxy of the original locations to estimate the spatial variance and correlation decay. The model was implemented in Fortran 90 code [21] developed by the authors.

Semi-variogram comparisons for this study suggested that samples of 150 locations preserve the original spatial correlation surface sufficiently, while smaller samples were unable to capture spatial range and variance simultaneously. We sampled different sets of locations between 50 and

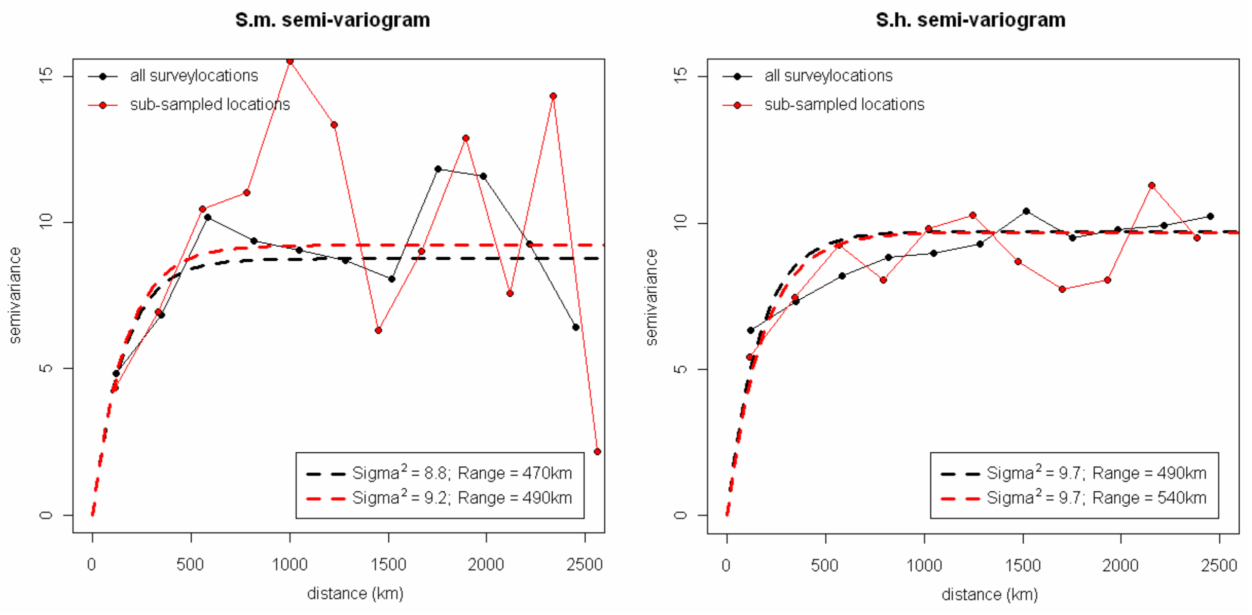

300 locations before the selection of the final sub-sample. The semi-variogram of each sample was compared with the semi-variogram of the complete dataset fitted via exponential correlation functions in R. The results indicated that samples of at least 150 locations sufficiently preserve the original correlation structure while samples with 50 or 100 locations fail. The semi-variogram based on the sub-sample of the 150 selected locations compared to the original set for each *Schistosoma* species is shown in the Figure.

Model validation

The performance of the models was assessed using model validation. A sample of 80% of the survey locations was employed as training set for model fit while the remaining 20% of the locations (test locations) were kept for model validation. The predicted outcomes at the k test locations are compared to the observed outcomes via three different approaches: ME, MAE, and BCI comparisons [16]. The ME shows the overall tendency of a model to over- or underestimate prevalence and it is calculated by $ME = \sum_{i=1}^{k} p_i - \hat{p}_i$, where p_i is the observed outcome and $\hat{p}_i$ the median of the predictions at test location i . The MAE provides information about the accuracy of a model based on the absolute distances between predictions and observations, $MAE = \sum_{i=1}^{k} \left| p_i - \hat{p}_i \right|$. The proportion of test locations being correctly predicted within the q -th BCI of the posterior predictive distribution (restricted by the lower centiles c_i^l and upper centiles c_i^u) is the outcome of the BCI approach, i.e., $BCI_q = \frac{1}{k} \sum_{i=1}^{k} \min\left(I\left(c_i^l < p_i\right), I\left(c_i^u > p_i\right)\right)$.

Additional references

44. Vincenty T (1975) Direct and inverse solutions of geodesics on the ellipsoid with application of nested equations. Surv Rev XXII.
45. Numerical Algorithms Group Ltd NAG Fortran Library. Oxford, UK: NAG. Available at: www.nag.co.uk/ (accessed: 22 August 2008).
46. Deville J, Tille Y (2004) Efficient balanced sampling: the cube method. Biometrika 91: 893-912.
47. R Development Core Team (2008) R: a language and environment for statistical computing. Vienna, Austria: R Foundation for Statistical Computing. Available at: http://www.R-project.org/ (accessed: 15 February 2010).
