# Supplementary material for: Geostatistical Model-Based Estimates of Schistosomiasis Prevalence among Individuals Aged ≤20 Years in West Africa
Source: PLoS Negl Trop Dis. 2011 Jun 14;5(6):e1194. doi: 10.1371/journal.pntd.0001194 (PMC3114755; doi:10.1371/journal.pntd.0001194)
Supplement: Checklist S1 — STROBE checklist. (PDF) [file pntd.0001194.s003.pdf]

STROBE Statement—checklist of items that should be included in reports of observational studies (see remark at page 2)

	Item No	Recommendation
**Title and abstract**	1	✓ (*a*) Indicate the study's design with a commonly used term in the title or the abstract LINE 35-36
		✓ (*b*) Provide in the abstract an informative and balanced summary of what was done and what was found LINES 35-51
**Introduction**		
Background/rationale	2	✓ Explain the scientific background and rationale for the investigation being reported 79-124
Objectives	3	✓ State specific objectives, including any prespecified hypotheses 125-131
**Methods**		
Study design	4	✓ Present key elements of study design early in the paper 135-143
Setting	5	✓ Describe the setting, locations, and relevant dates, including periods of recruitment, exposure, follow-up, and data collection 135-160
Participants	6	✓ (*a*) *Cohort study*—Give the eligibility criteria, and the sources and methods of selection of participants. Describe methods of follow-up<br>*Case-control study*—Give the eligibility criteria, and the sources and methods of case ascertainment and control selection. Give the rationale for the choice of cases and controls<br>*Cross-sectional study*—Give the eligibility criteria, and the sources and methods of selection of participants 139-153
		/ (*b*) *Cohort study*—For matched studies, give matching criteria and number of exposed and unexposed<br>*Case-control study*—For matched studies, give matching criteria and the number of controls per case
Variables	7	✓ Clearly define all outcomes, exposures, predictors, potential confounders, and effect modifiers. Give diagnostic criteria, if applicable 145-160, 163-198, 206-239
Data sources/ measurement	8*	✓ For each variable of interest, give sources of data and details of methods of assessment (measurement). Describe comparability of assessment methods if there is more than one group 163-203
Bias	9	✓ Describe any efforts to address potential sources of bias 145-160, 206-239
Study size	10	✓ Explain how the study size was arrived at Fig 1, 141-153
Quantitative variables	11	✓ Explain how quantitative variables were handled in the analyses. If applicable, describe which groupings were chosen and why 206-224, 169-185
Statistical methods	12	✓ (*a*) Describe all statistical methods, including those used to control for confounding 206-239
		/ (*b*) Describe any methods used to examine subgroups and interactions
		✓ (*c*) Explain how missing data were addressed 154-160
		/ (*d*) *Cohort study*—If applicable, explain how loss to follow-up was addressed<br>*Case-control study*—If applicable, explain how matching of cases and controls was addressed<br>*Cross-sectional study*—If applicable, describe analytical methods taking account of sampling strategy
		✓ (*e*) Describe any sensitivity analyses 235-239

Continued on next page

**Results**		
Participants	13* ✓	(a) Report numbers of individuals at each stage of study—eg numbers potentially eligible, examined for eligibility, confirmed eligible, included in the study, completing follow-up, and analysed Fig 1, 243-246
	✓	(b) Give reasons for non-participation at each stage Fig 1
	✓	(c) Consider use of a flow diagram Fig 1
Descriptive data	14* ✓	(a) Give characteristics of study participants (eg demographic, clinical, social) and information on exposures and potential confounders 246-252, Fig 2, Fig 3, Table 2
		(b) Indicate number of participants with missing data for each variable of interest no missings
		(c) *Cohort study*—Summarise follow-up time (eg, average and total amount)
Outcome data	15*	*Cohort study*—Report numbers of outcome events or summary measures over time
	✓	*Case-control study*—Report numbers in each exposure category, or summary measures of exposure
		Cross-sectional study—Report numbers of outcome events or summary measures Table 2
Main results	16 ✓	(*a*) Give unadjusted estimates and, if applicable, confounder-adjusted estimates and their precision (eg, 95% confidence interval). Make clear which confounders were adjusted for and why they were included Table 2-5, 253-259, 279-307
		(*b*) Report category boundaries when continuous variables were categorized Table 3-4
		(*c*) If relevant, consider translating estimates of relative risk into absolute risk for a meaningful time period
Other analyses	17 ✓	Report other analyses done—eg analyses of subgroups and interactions, and sensitivity analyses 310-317
**Discussion**		
Key results	18 ✓	Summarise key results with reference to study objectives 320-326, 365-386
Limitations	19 ✓	Discuss limitations of the study, taking into account sources of potential bias or imprecision. Discuss both direction and magnitude of any potential bias 387-478
Interpretation	20 ✓	Give a cautious overall interpretation of results considering objectives, limitations, multiplicity of analyses, results from similar studies, and other relevant evidence 327-386
Generalisability	21 ✓	Discuss the generalisability (external validity) of the study results ~~479~~ 327-356, 365-386
**Other information**		
Funding	22 ✓	Give the source of funding and the role of the funders for the present study and, if applicable, for the original study on which the present article is based ~~[illegible]~~ Entered in PLOS NTDs submission system.

*Give information separately for cases and controls in case-control studies and, if applicable, for exposed and unexposed groups in cohort and cross-sectional studies.

Note: An Explanation and Elaboration article discusses each checklist item and gives methodological background and published examples of transparent reporting. The STROBE checklist is best used in conjunction with this article (freely available on the Web sites of PLoS Medicine at http://www.plosmedicine.org/, Annals of Internal Medicine at http://www.annals.org/, and Epidemiology at http://www.epidem.com/). Information on the STROBE Initiative is available at www.strobe-statement.org.

REMARK: We used compiled survey data, therefore not all points listed here match well to our manuscript. However, we tried to identified equivalent answers in our manuscript.
